# Monomorphic Ventricular Tachycardia Originating From Right Ventricular Outflow Tract as a Trigger for the Recurrent Ventricular Fibrillation in a Patient With Brugada Syndrome

**DOI:** 10.5812/cardiovascmed.17113

**Published:** 2014-04-01

**Authors:** Mohammadali Akbarzadeh, Majid Haghjoo

**Affiliations:** 1 Cardiac Electrophysiology Research Center, Rajaie Cardiovascular Medical and Research Center, Iran University of Medical Sciences, Tehran, IR Iran

**Keywords:** Brugada Syndrome, Catheter Ablation, Tachycardia

## Abstract

**Introduction:**

Brugada Syndrome is a cardiac ion channel disorder that affects the sodium current. This syndrome is characterized by cove-shaped ST elevation in ECG leads V1 to V3 in the absence of structural heart disease.

**Case Presentation:**

A 36-year-old man diagnosed with Brugada Syndrome was reffered to our center with frequent implantable cardioverter-defibrillator (ICD) discharges. ICD interrogation showed several appropriate ICD intervention for tachycardia detected in the ventricular fibrillation zone. Unfortunately, quinidine was not available in our country at the time of admission; therefore, we decided to ablate suspicious arrhythmogenic substrates. Programmed ventricular stimulation from right ventricle (RV) reproducibly induced a sustained ventricular tachycardia with left bundle branch block morphology and inferior axis. RV outflow tract (RVOT) endocardially mapped and earliest activation signal (90 milliseconds) achieved at posterior aspect of the RVOT septum. RF energy application at that site terminated the tachycardia and no inducible tachycardia was detected. During two-year follow-up, he had no episodes of ICD therapy and remained symptom-free with any antiarrhythmic drug.

**Discussion:**

This case clearly indicated that catheter ablation might be considered as a viable option in every patient with Brugada syndrome and frequent ICD discharge. During the electrophysiology study, intravenous procainamide may also be used to reveal future arrhythmogenic focus in this group of patients.

## 1. Introduction

Brugada Syndrome (BrS) is a cardiac ion channel disorder that affects the sodium current. This syndrome is characterized by cove-shaped ST elevation in ECG leads V1 to V3 (spontaneously or after drug challenge) in the absence of structural heart disease or other causes. Patients are at increased risk of syncope and sudden cardiac death (SCD) due to polymorphic ventricular tachycardia (VT) or ventricular fibrillation (VF). Prolongation of PR interval, P wave abnormalities, slight QT prolongation, sick sinus syndrome, and atrial fibrillation were also described with this syndrome. The occurrence of monomorphic VT is extremely rare (1, 2).

## 2. Case Presentation

**Figure 1.**
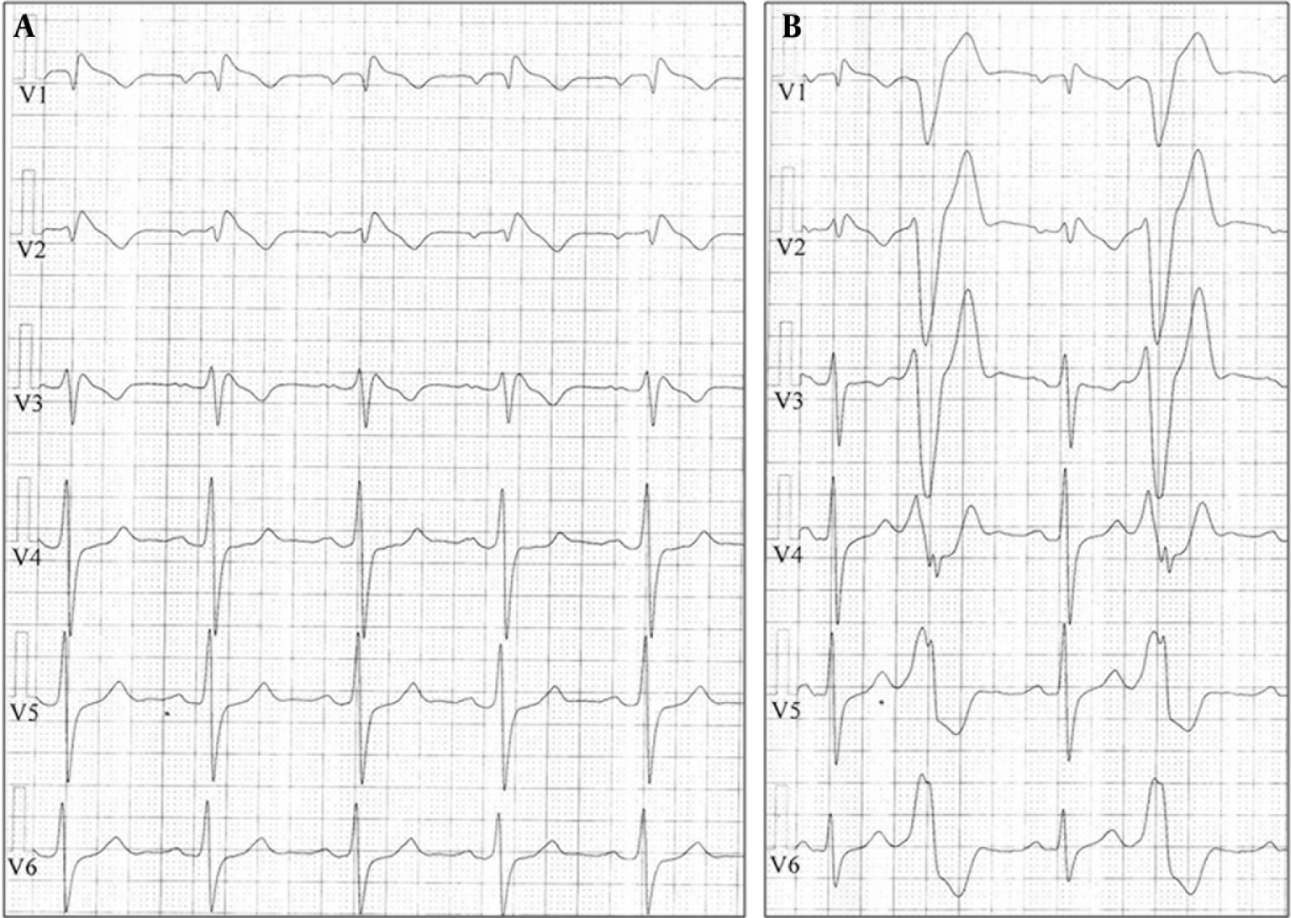
Typical Brugada ECG Pattern Induced by Intravenous Procainamide (Panel A); Observed PVC Bigeminy With LBBB Pattern and Inferior Axis During the Test (panel B) LBBB, left bundle branch block; PVC, premature ventricular complex.

A 36-year-old man, without any structural heart disease, was referred to our hospital due to frequent episodes of palpitations and syncope. The 12-lead ECG during sinus rhythm showed right bundle branch block morphology, ST segment elevation with saddle back configuration in lead V2, and normal QTc interval. After admission, episodes of sustained atrial flutter were detected. During electrophysiology study, a sustained typical atrial flutter was induced. The study also revealed a prolonged corrected sinus node recovery time (1300 msec). A bidirectional block was created across the cavotricuspid isthmus following linear irrigated radiofrequency (RF) ablation. Drug challenge test with intravenous procainamide resulted in cove-shaped ST segment elevation in the right precordial leads followed by emergence of premature ventricular complex (PVC) bigeminy with left bundle branch block (LBBB) morphology, inferior axis, and QRS transition at V4 (Figure 1). The presence of late potentials was detected by signal-averaged ECG. Patient refused implantable cardioverter defibrillator (ICD) implantation at that time.

**Figure 2.**
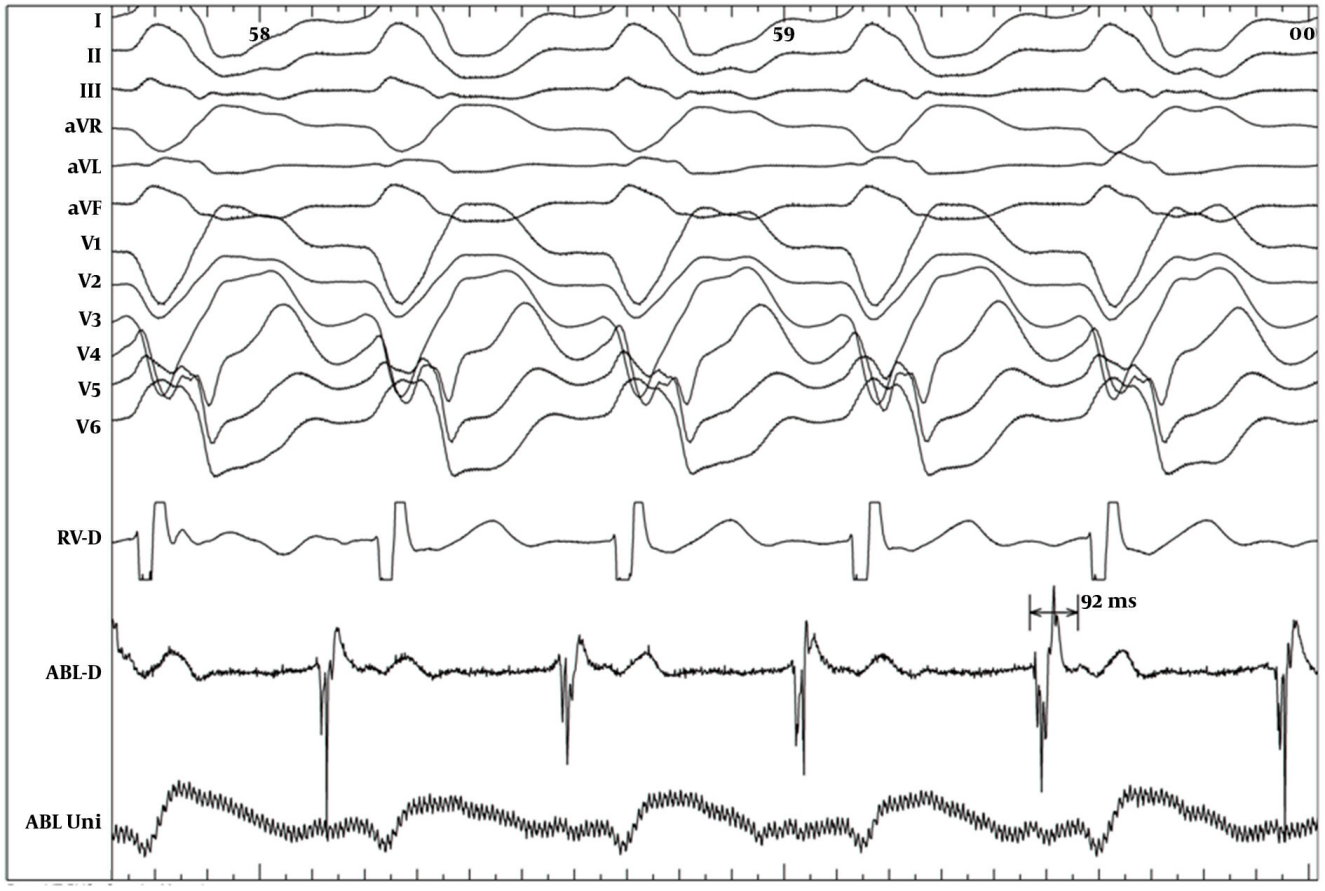
Intracardiac Recording During Monomorphic VT Tachycardia with LBBB Pattern and Inferior Axis and a Very Early Signal (92 msec Earlier Than Surface QRS) Detected in the Posterior Part of the Septal RVOT RVOT, right ventricular outflow tract; VT, ventricular tachycardia; LBBB, left bundle branch block.

**Figure 3.**
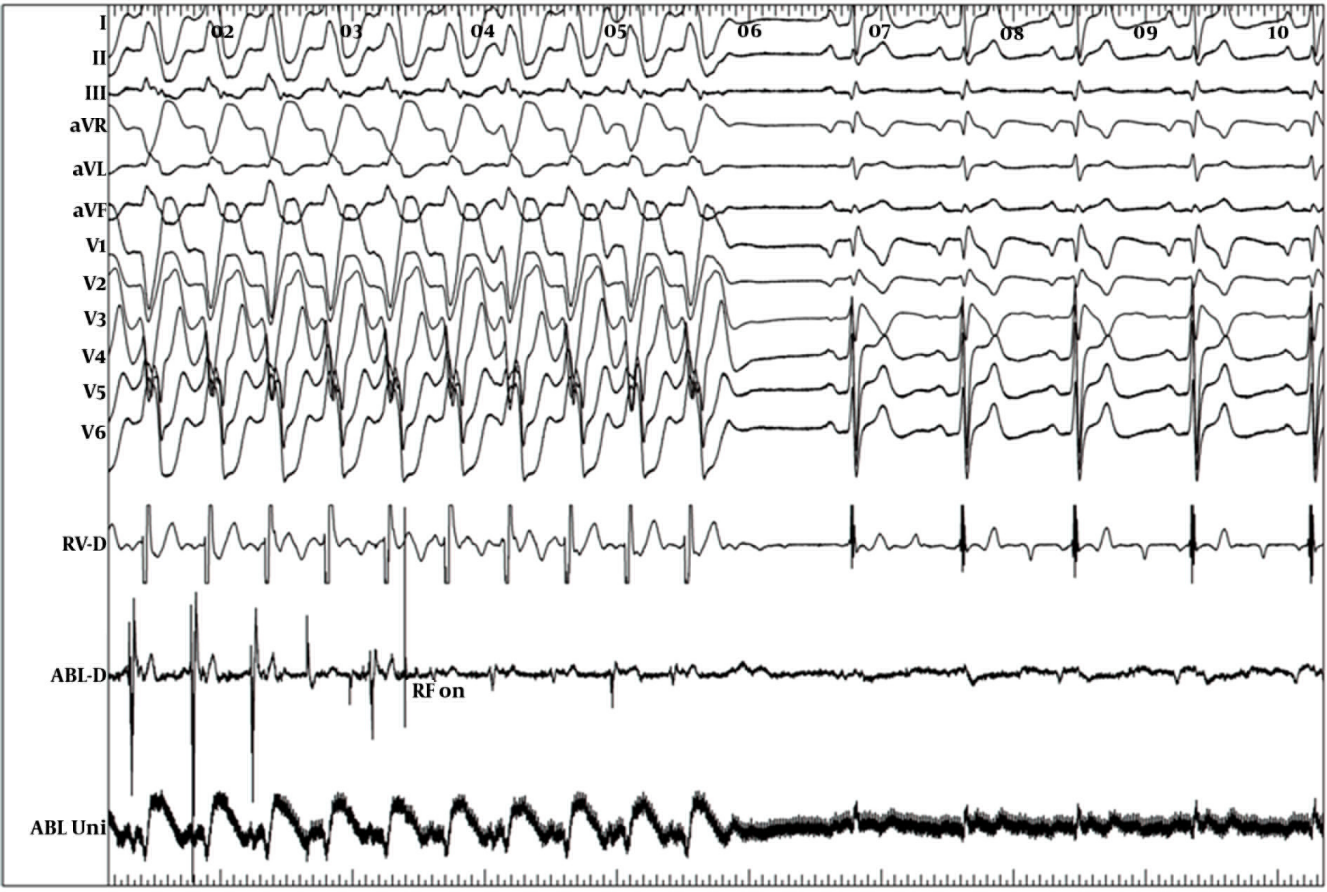
Tachycardia Termination by Radiofrequency Energy Application at the Site of Early Signal

One year later, he was admitted again due to exacerbation of the palpitation and recurrence of syncope. On admission, sinus bradycardia (35 beat/min) was detected in ECG. In this admission, a dual-chamber ICD was implanted (Protecta DR, Medtronic Inc., Minneapolis, MN, USA). ICD was programmed with a single VF detection zone (200 beat/min) and a VT monitoring zone (150 beat/min. During follow-up, patient received frequent episodes of appropriate shocks due to reverse VF. In addition, frequent episodes of slower VT at the range of monitoring zone were detected. Unfortunately, quinidine was not available in our country at that period; therefore, we decided to try ablation of suspicious arrhythmogenic substrates. Patient was brought to the catheterization room for the electrophysiological study (EPS). During EPS patient developed hemodynamic stable monomorphic sustained VT with LBBB pattern and inferior axis (cycle length 460 milliseconds) similar to morphology of the PVCs induced during procainamide test. Right ventricular outflow tract (RVOT) endocardially mapped and earliest activation signal (90 msec) achieved at posterior aspect of the RVOT septum (Figure 2). RF energy application at that site terminated the tachycardia and no tachycardia was inducible (Figure 3). During two-year follow-up, he had no episodes of ICD therapy and remained symptom-free with any antiarrhythmic drug.

## 3. Discussion

In this paper, we described a case of BrS with the history of atrial flutter ablation and sick sinus syndrome that had recurrent episodes of VF causing ICD discharges. Moreover, he had frequent episodes of monomorphic VT with LBBB morphology and inferior axis. According to our knowledge, monomorphic VT was reported in less than 15 cases of BrS or Brugada-like syndromes up to this date (2). ICD implantation is recommended in BS patients with history of cardiac arrest, syncope, or documented ventricular arrhythmia; however, the only useful drug to treat recurrent ICD shock due to VF is Quinidine, which is not available in many countries (3, 4). The role of the RF catheter ablation has not been evaluated adequately in BS. Haissaguerre et al. (5) reported triggering role of PVCs in the initiation of VF and preventing recurrence of syncope or SCD with focal ablation of these PVCs. Recently, Nademanee et al. (6) showed that catheter ablation over the anterior aspect of RVOT, where the abnormal delay depolarization recorded, not only normalized the Brugada ECG pattern but also prevented VT or VF recurrences in these patients.

In our case, we could not document whether VF episodes were triggered by premature beats, which were induced in procainamide test or during monomorphic VT, or this monomorphic RVOT VT was coexisted incidentally with BrS. However, complete prevention of the ICD shocks indicated that some episodes of RVOT VT were degenerated to VF and were treated by ICD. This case clearly indicated that catheter ablation might be considered as a viable option in every patient with BrS and frequent ICD discharge. During EPS, intravenous procainamide may also be used to reveal future arrhythmogenic focus of VT/VF in this group of the patients.
